# Improving the Mechanical Strength of Ductile Cast Iron Welded Joints Using Different Heat Treatments

**DOI:** 10.3390/ma12142263

**Published:** 2019-07-14

**Authors:** Eva S.V. Marques, F.J.G. Silva, Olga C. Paiva, António B. Pereira

**Affiliations:** 1 ISEP—School of Engineering, Polytechnic of Porto, 4249-015 Porto, Portugal; 2 TEMA—Centre for Mechanical Technology and Automation, Department of Mechanical Engineering, University of Aveiro, Campus de Santiago, 3810-193 Aveiro, Portugal

**Keywords:** high strength cast iron, nodular cast iron, shield metal arc welding, heat-affected zone, phases, mechanical strength, hardness, welding, welding cast iron, microstructure

## Abstract

The main advantage of welding cast iron is to recover parts by repairing defects induced by casting processes (porosities, etc.), before they enter their working cycle, as well as repair cracks or fractures when already in service. This method contributes to decreased foundry industrial waste and avoids the additional energy costs of their immediate recycling. Therefore, it is necessary to have a welded joint with similar or better characteristics than the parent material. The major problem of welding cast iron is that this material has a very high content of carbon in comparison to steel (≈3%). Therefore, when it is heated by the very high temperatures from arc welding and during its process of solidification, very hard and brittle phases originate, known as ledeburite and martensite, and appear in the partially melted zone and in the heat-affected zone. Eventually, this problem can be solved by implementing heat treatments such as preheat or post weld heat treatments under specific parameters. Therefore, in this study, the aim is to collect data about the effects of heat treatments performed at different temperatures on welded joints of high strength ductile cast iron (SiboDur^®^ 450), and to evaluate the effects of heat treatments performed at diverse temperatures on welded joints of this type of material, using Shield Metal Arc Welding and nickel electrodes. Mechanical strength, hardness, and microstructure were analyzed, showing that the best mechanical strength in the joint (380 MPa) was obtained using two passes of E C Ni-Cl (ISO EN 1071:2015) filler metal and post weld heat treatments (PWHT) of 400 °C for two hours.

## 1. Introduction

Cast iron is a class of Fe-C alloys with higher C content than steels and, depending on the alloying elements and the microstructure formed during solidification, it is classified as lamellar /grey cast iron, nodular/ductile cast irons or white cast iron. Some of these cast irons can still be subjected to specific heat treatments, which give rise to other classes of cast iron, such as malleable or austempered ductile iron (ADI) [1].

Ductile cast iron (DCI) is a cast iron type which presents properties as good as some kinds of steels, such as mechanical strength, fatigue and wear resistance, and machinability. DCIs are used in diverse applications, for example, internal combustion blocks, clutches, pistons, and automotive chassis parts [1,2]. 

In addition, DCIs have a great number of advantages as compared with other cast irons and common steels, such as obtaining complex shapes through casting and foundry processes and have lower specific weights allowing casting of thinner-walled parts while maintaining superior mechanical properties (ductility and fracture toughness) [3,4]. 

Most of the properties of DCIs emerge from their microstructure, which depends on several characteristics such as chemical composition, process parameters, and inoculation type used during their production [5,6,7]. Moreover, DCIs originate from the melting of a metallic material such as steel, and then, its weight defines its chemical composition, which will help in the other additive element choice. Inoculation is usually performed immediately before casting by adding Mg (with a residual content of 0.04%) or Ce [8] which gives rise to the nodularization of the graphite. Nodular graphite allows DCIs to have mechanical properties superior to other cast irons such as higher toughness and greater resistance to plastic deformation [8].

Commonly, DCIs have a carbon equivalent value (CEV) between 2.0% and 4.0%, and therefore the volume of the graphite precipitated during the solidification completely secures all the sections of the mold. Other relevant elements are Si (2% to 2.8%), Mn (0.1% and 1.0%), Cr (<0.08%) due to its carbonic nature, and Pb (<0.008%) as it deforms the spherical shape of the graphite [9]. It is also possible to detect small amounts of P and S [8], which are the main cause of problems during production and processing DCIs as they affect the material’s mechanical properties by promoting the appearance of prejudicial structures, such as carbides and/or irregular graphite [10,11,12,13].

Some elements of the chemical composition of DCIs influence their own weldability, for example, the Si content, since it is a constituent known for its high graphitization. This has the tendency to increase austenite/graphite eutectic temperature and to lower the eutectic austenite/cementite temperature. In order to avoid the formation of cementite, the Si content of DCIs must be as high as the specification allows it to be [14,15]. Other elements to consider are Mn and Ni, where the higher the content of these, the greater the difficulties in welding and the greater the need for preheating treatments [14]. It is also important to control the P content, since it contributes to the formation of eutectic Fe-Fe3P, steadite, in the grain boundaries, which contributes to cracks caused by contraction and cooling of the material. As this compound has a low melting point, this may cause hot cracking in the weld bead or in the heat-affected zone (HAZ) area [14,15]. The element S causes brittle fractures when there are high levels of Ni. A high content of O and S accelerate the precipitation of carbides which, consequently, can also cause cracking of the joint [14].

The practice of welding DCIs is not common, even in the metalworking industry, due to problems related to its chemical composition (high amount of carbon) [16], which promotes martensite and carbides formation in the HAZ and the partially melted zone (PMZ). Other problems are related to its low ductility which does not accommodate residual stresses in the welded joint, causing fractures. In addition, the high content of P, S, and O, can lead to pores in the welded joint. Other problems associated with welding DCIs are related to their own processing method, for example, defects in casting, such as sand inclusions and contraction. These defects do not let the total fusion between the parent material and the filler metal occur [14].

Despite these challenges, welding processes are mainly used as corrective maintenance and are applied to repairing casting defects in areas with no functional requirements [17,18]. The Shield Metal Arc welding (SMAW) process is one of the most exploited for welding DCIs [19] since it has easier maneuverability, easier access of filler metal (electrodes), and it has a low welding speed which can contribute to lower cooling rates. 

There are generally three types of electrodes used for welding DCIs: cast iron filler metal, Ni, and Ni-Fe filler metal alloy [20]. Electrodes are considered to be pure Ni and may cause the carbon of the parent material to dissolve in fine graphite in the welded joint, making it ductile, and consequently, machinable. In addition, Ni suppresses the diffusion of carbon in the interface between the parent material and the filler metal, reducing the formation of carbides and other harmful structures with high hardness such as martensite in the HAZ and PMZ areas. This occurs because Ni has a low coefficient of heat release, which helps the weld joint to have a slow cooling rate, [14] as shown in the Pouranvari experiments [21]. The Ni-Fe electrodes were also studied and compared with pure Ni electrodes by Pascual et al. [16], and it was concluded that the high purity Ni electrode had better results than the Ni-Fe electrodes. Therefore, welds performed with Ni electrodes have been given greater importance [22,23] due to the microstructure achieved during welding processes [24].

Several studies have been accomplished in order to find the best results for mechanical properties of welded DCIs [16,19,22,25,26]. Heat treatments are a common way to surpass the usual challenges encountered when welding DCIs [16,25,27,28]. The preheating temperature depends on the chemical composition or CEV of the alloy, which conditions the hardenability of the DCI [20]. The hardness of the HAZ can be limited using preheating treatments, followed by a slow cooling rate after welding. Preheating reduces the cooling rate of the filler metal in the HAZ, which, consequently, decreases the amount of martensite and cementite, which create the hardness in these areas [29]. It also prevents cold cracking, reduces residual stress and distortion, and improves the fluidity of the material [14,16,20]. The minimum preheating temperature required to avoid the formation of martensite should be above the formation temperature of its own (Ms temperature), 230 °C. The definition of the preheating temperature depends on the thickness of the parts, as well as the thermal energy of the process [14]. The required temperatures for preheating are the following [14,15,16]: 250 °C and 400 °C, which are suitable for cast iron where the percentage of C is equal to or greater than 3%; 300 °C, where the transformation of austenite to fine perlite may occur; 425 °C, which can be used to prevent the formation of martensite, but it is not advisable, since it may give rise to cementite in the HAZ; 500 °C, the temperature range which forms a continuous net of cementite in the melting lines; 600 °C and 650 °C, which are suitable temperatures when there is a lot of heat dissipation in the workpiece, and finally, 760 °C, which is the maximum recommended temperature for preheating, since this temperatures is close to the critical temperature of cast iron (790 °C). 

Post weld heat treatments (PWHT) can lower the hardness of the HAZ but do not restore ductility and toughness to its own original values, due to the formation of a fine dispersion of secondary graphite that accompanies the decomposition of martensite. This structure in the HAZ can be tempered to a structure with less hardness [14]. The PWHT is usually advisable for the following reasons [30,31]: It improves the ductility and the machinability of the HAZ and the joint, it decomposes the carbides formed during welding processes, it transforms martensite into a less brittle structure, and it relieves residual stresses formed during the welding cycles. It is also possible to produce a similar effect to the PWHT by performing further welding passes, relative to the former ones in the welded joint. 

Manifold studies about the influences of heat treatments (preheating and PWHT) were carried out by Ebrahimnia et al. [19] and El-Banna [17,27]. These studies showed good results in terms of improvement of UTS (Ultimate Tensile Strength) values, by subjecting DCIs to several preheating temperatures such as 200 °C, 300 °C, and 400 °C. Askari-Paykani et al. [25], Mandal [26] and Connor [32], studied the influence of cooling rates, it was referred by [25] that when preheating was used for DCI welding, cooling conditions did not present a significant influence on the UTS value of the joined part. However, [26,32] believe that rapid cooling rates can increase the probability of martensite appearance in the HAZ. 

This work intends to estimate the effects of heat treatments performed at different temperatures (in the range of 300 °C to 700 °C), through the implementation of several thermal cycles, namely, preheating, PWHT or both procedures, on welded joints of SiboDur® 450, by the SMAW process and using 98% Ni electrode. This work is an improvement from one previously elaborated [33] in this article. 

## 2. Materials and Methods

The main objective of this work is to determine which heat treatment is the best to improve the mechanical properties of the welded joint. To measure this property in the experimental samples, tensile tests were performed following the international standard [34]. Since the aim of this work is to achieve a UTS value of 360 MPa, which corresponds to an 80% mechanical strength of the parent material, tensile tests are of utmost importance, due to the fact it is the first stage to exclude or accept the sets of experimental samples. Procedures such as metallographic analyses [35] and hardness tests [36] were performed only with the intent to support possible conclusions of this work.

### 2.1. High Strength Ductile Cast Iron—SiboDur^®^ 450

With the evolution of the manufacturing technology, there have been many newer materials, among them is a DCI called SiboDur^®^ 450. This material is a high strength ductile cast iron, with composition and characteristics close to the EN-GJS-450-10 (DIN EN 1563:2012) [37], developed and industrialized by the company Georg Fischer (GF), with a superior mechanical strength (450 to 700 MPa [38]) and an elongation rate up to 22% as compared with regular cast irons. GF provided all raw materials to produce the samples which were used in this study. Table 1 shows the main chemical composition of SiboDur^®^ 450 obtained from the material datasheet [38]. 

**Figure 1:**
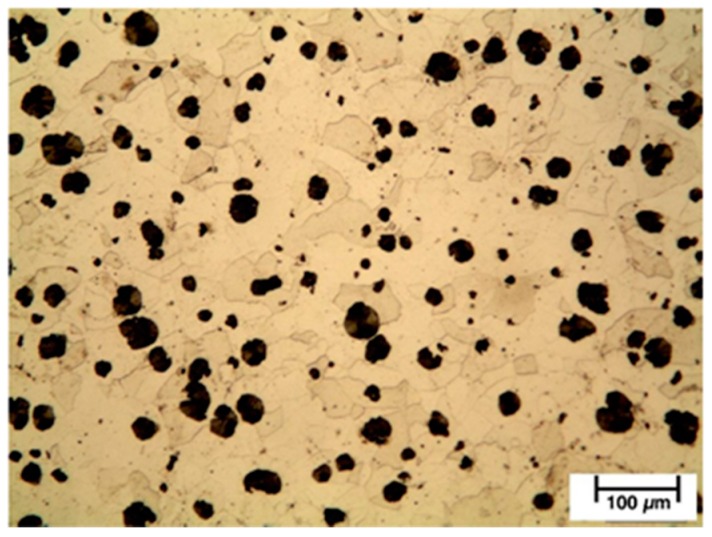
Microstructure of Sibodur^®^ 450.

In a raw state (without heat treatments), SiboDur^®^ 450 presents a pearlitic matrix microstructure with a ferrite content of ≤20% (Figure 1) and a UTS value of 450 MPA. As well, this material presents Young’s modulus of 170 GPA, hardness between 150 HBW and 190 HBW, and a thermal expansion coefficient of 12.2 × 10^−6^/K.

### 2.2. Sample Preparation

The raw material was received as rods. Although manufacturing samples, it is possible that the process might slightly affect the surface quality of them, as well as the tensile strength properties (less than 5%). The general dimension of the rods was 190 mm in length with three different diameters: D1 = 45 mm, D2 = 36 mm, and D3 = 38 mm [33]. The experimental samples for welding were cut by saw and CNC machined in order to obtain nine test specimens from each rod received.

### 2.3. Heating Cycle Parameters

In the literature, it is quoted that there are three heat treatments available that can be applied in welded DCIs: Preheating (before welding), PWHT (after welding), or both. Preheating is generally used to raise and normalize the sample temperatures before welding. Since the thickness of the weld samples were 4 mm, a preheat temperature of 300 °C for two hours was enough to homogenize the test samples, as shown in previous studies. 

PWHT was also applied to some test samples at the following temperatures: 400 °C, 500 °C, and 700 °C for two hours. When conjugating preheating with PWHT, the samples were preheated at 300 °C for one hour, and then, after welded, each set was submitted to heat treatment at temperatures of 300 °C, 400 °C, and 500 °C, respectively, for two hours. A Nabertherm N 11/H muffle furnace (Nabertherm GmbH, Lilienthal, Germany) was used for these procedures.

### 2.4. Welding Parameters

The process chosen to weld the samples was SMAW. This process presents a low welding speed which is an important factor as this contributes to lower cooling rates. The welding equipment used was an OERLIKON SAXOTIG 1600 welder (OERLIKON, Pfäffikon, Switzerland). The filler metal (FM) chosen was a 98% Ni electrode with a diameter of 2.5 mm (E C Ni-Cl (EN ISO 1071:2015 [39]). The electrode was connected to the positive pole of the machine and was combined with an amperage of 60 A, 70 A, and 80 A, depending on the heat treatment that was performed (these values were recommended by the electrode manufacturer). 

**Figure 2:**
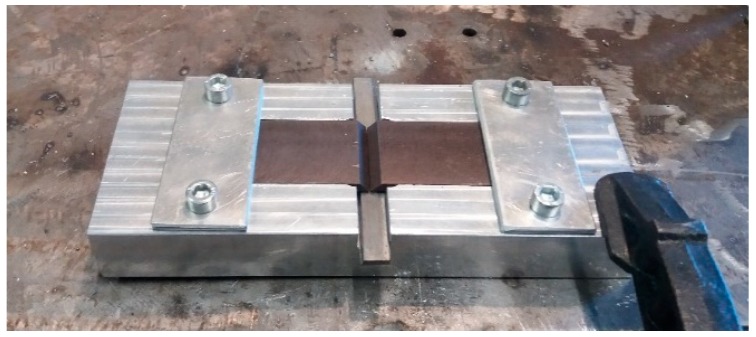
Jig designed and manufactured for welding Sibodur^®^ 450 samples in this work.

To avoid defection and distortion of the samples, a jig was designed and manufactured to be a support while welding. The jig is presented in Figure 2.

### 2.5. Sample Reference Coding and Welding Parameters Used in the Experiments

As the necessary number of experimental samples to consider was high, i.e., a total of 33, a coding sequence was created in order to obtain an identification of each sample with their own different welding parameters: Number of weld beads (two or four) and heat treatment (pre-/post weld heating cycles or both). Each sample was identified with the following sequence:

- N—sample number;
- Px—preheating cycle temperature (°C);
- Wz—number of weld beads;
- Py—post weld heat cycle temperature (°C).

Table 2 demonstrates the differences between the welding procedures and heat cycles parameters used in this study.

### 2.6. Analysis of Mechanical Properties

#### 2.6.1. Tensile Properties 

**Figure 3:**
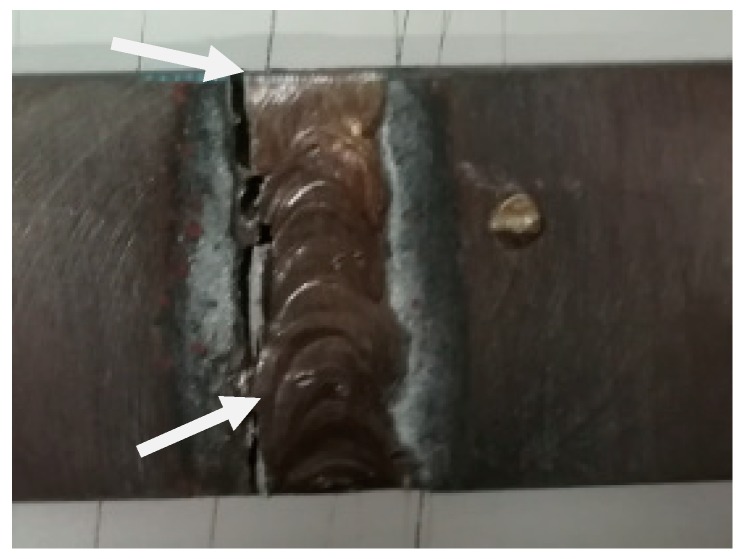
Example of sample failure in the heat-affected zone (HAZ) area found during tensile tests.

The main reason for tensile tests was to evaluate the mechanical strength properties of the samples in areas such as parent metal, HAZ, PMZ, and filler metal, which allowed determination of the weakest joining point. These tests were performed using a universal Shimadzu Autograph AGS-X-100 kN machine (Shimadzu, Kyoto, Japan) following the ISO 4136:2012 [35] standard. The speed during the tensile tests was kept constant at 2 mm/min, using a distance of 75 mm between the clamps. A fracture originated during this test and is shown in Figure 3.

#### 2.6.2. Metallographic Analysis

The metallographic analysis revealed characteristics on a macro- and microscopic level, showing, for example, microstructural changes that occurred during welding. In addition, the quality of the welded zone was evaluated in terms of homogeneity, defects such as cracks, pores or inclusions, etc. The necessary procedure to perform this destructive testing method is described in [33].

Experimental samples were then analyzed on an OLYMPUS BX51M optical microscope (Olympus, Tokyo, Japan) and on a SEM (scanning electron microscopy) microscope FEI QUANTA 400 FED equipped with an EDS (energy-dispersive X-ray spectroscopy) system (FEI, Hillsboro, OR, USA). These procedures were completed following the ISO 17639:2003 [35] international standard. 

#### 2.6.3. Hardness Analysis

The experimental samples for the metallographic analysis were the same as those used in the hardness tests. Following the ISO 9015-1:2011 [36] standard, this destructive testing method allowed the examination of hardness profiles of the welded joints by measuring their surface hardness. A row of indentations was performed, respecting the minimum distance required by the standard between indentations (0.7 mm), to determine the evolution of surface hardness through the welded joint, from the parent metal in one side to the parent metal in the other side of the weld, crossing over the weld and HAZ areas. In order to carry out these tests, an EMCO-TEST M4U 025G3 durometer (EMCO-TEST PRÜFMASCHINEN GMBH, Kuchl, Austria) was used, which was equipped with a pyramidal diamond indenter (136° angle between faces).

## 3. Results

### 3.1. Tensile Test Results

The obtained results from the tensile tests are of utmost importance, as it is the primary criteria for selection of the best samples. Even though the welded joints have lower strength than the parent material, a UTS of 360 MPa or higher (corresponding crudely to 80% of the material’s original UTS of 450 MPa) was an acceptable value for a welded ductile cast iron. Therefore, results below 360 MPa were excluded as they did not fulfil the previously established rule. Through the verification of Table 2, this rule eliminates four groups of samples: P300W2P0, P0W2P700, P300W2P400, and P300W2P500. The summary of tensile test results is shown in Table 2. The first row corresponds to experiments carried out over the parent material samples without any kind of heat treatment.

### 3.2. Metallography Analysis Results

**Figure 4:**
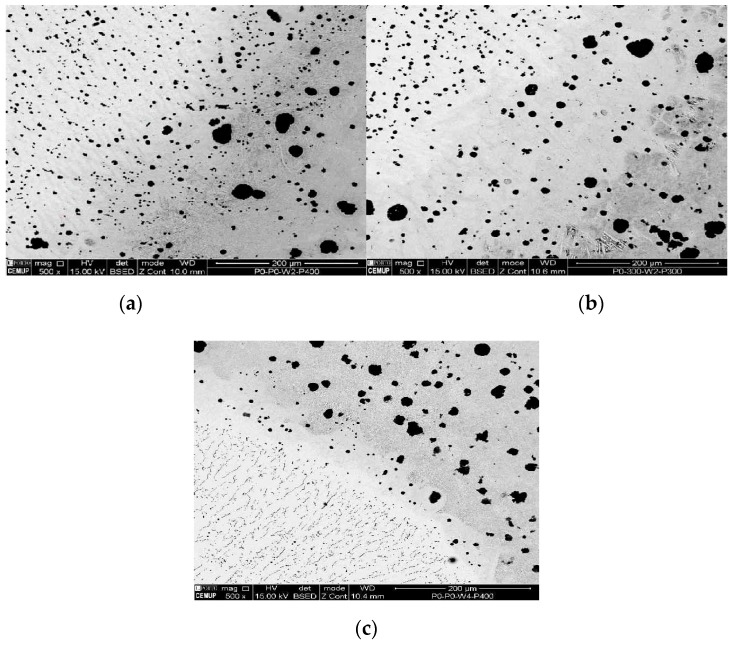
Scanning electron microscopy (SEM) analysis of the experimental samples etched with 4% Nital and 500× magnification: (**a**) sample 1P0W2P400 (UTS 390.0 MPa), (**b**) sample 2P300W2P300 (UTS 394.2 MPa), and (**c**) sample 1P0W4P400 (UTS 394.0 MPa).

**Figure 5:**
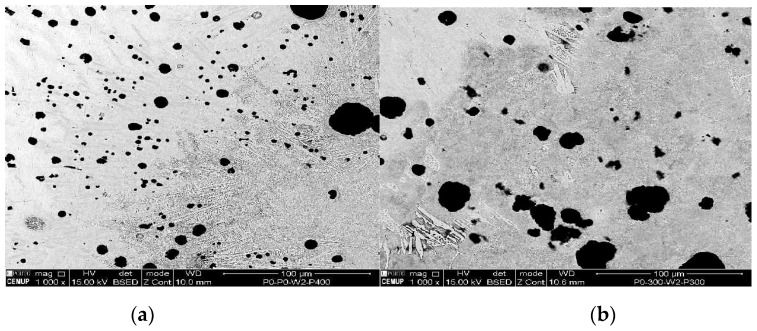

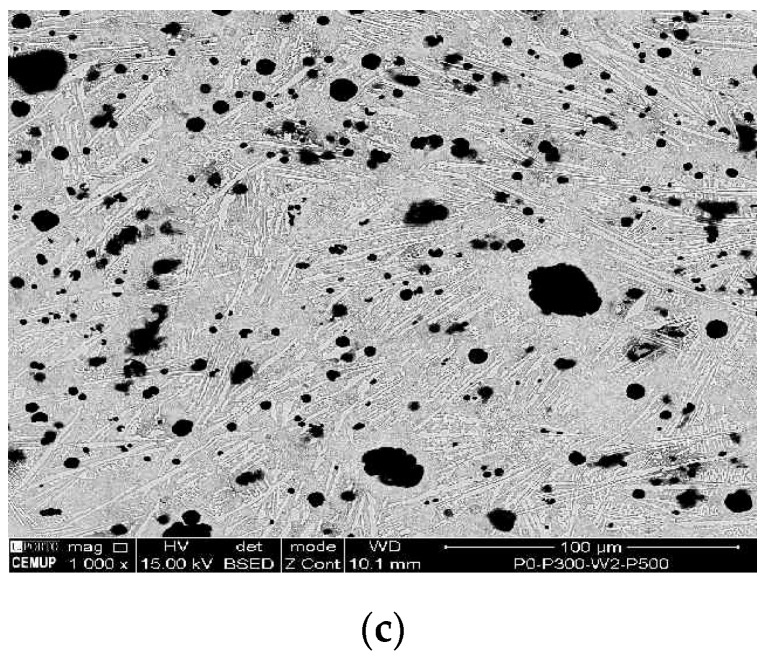
SEM analysis of the experimental samples etched with 4% Nital and 1000× magnification: (**a**) sample 1P0W2P400 (UTS 390.0 MPa), (**b**) sample 2P300W2P300 (UTS 394.2 MPa), and (**c**) sample 1P0W4P400 (UTS 394.0 MPa).

**Figure 6:**
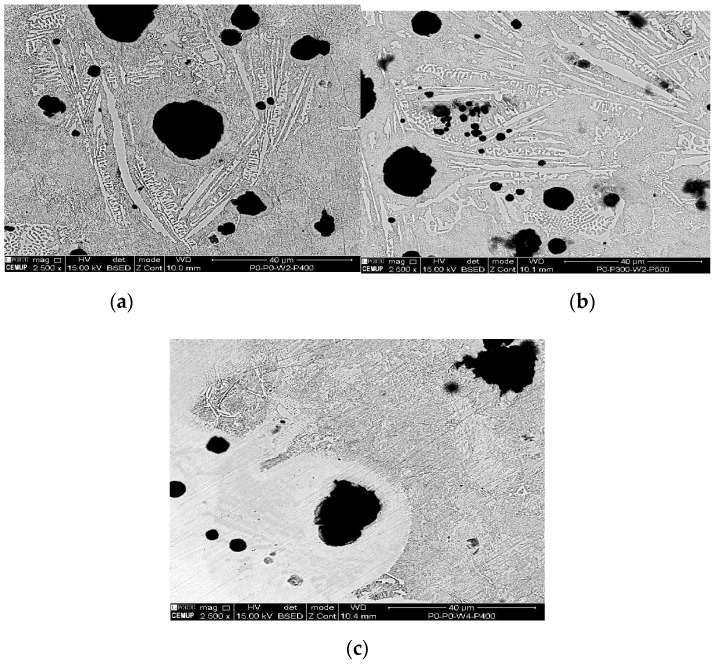
SEM analysis of the experimental samples etched with 4% Nital and 2500x magnification: (**a**) sample 1P0W2P400 (UTS 390.0 MPa), (**b**) sample 2P300W2P300 (UTS 394.2 MPa), and (**c**) sample 1P0W4P400 (UTS 394.0 MPa).

**Figure 7:**
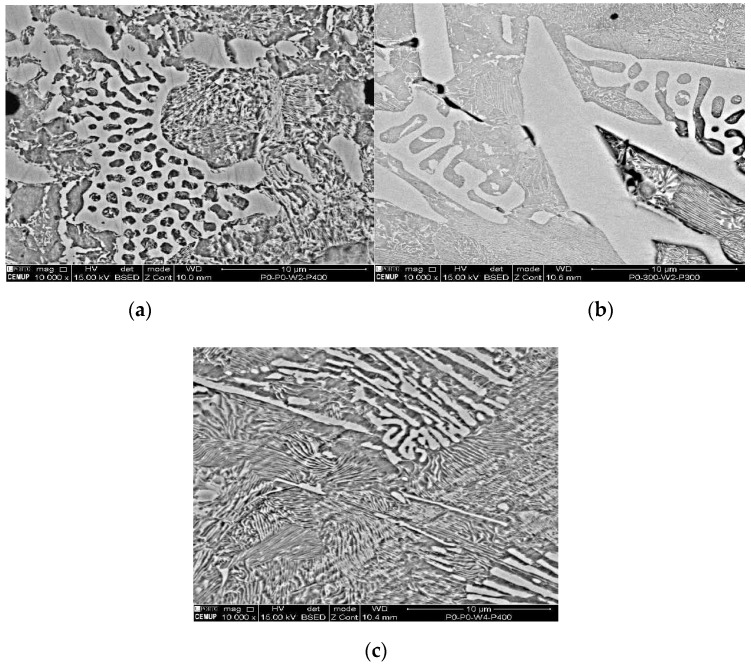
SEM analysis of the experimental samples etched with 4% Nital and 10000x magnification: (**a**) sample 1P0W2P400 (UTS 390.0 MPa), (**b**) sample 2P300W2P300 (UTS 394.2 MPa), and (**c**) sample 1P0W4P400 (UTS 394.0 MPa).

The tested samples that obtained the uppermost UTS values were chosen to be analyzed by metallography analysis. The sets of samples selected were: P0W2P400, P0W4P400, and P300W2P300. On the basis of the microscopic analysis some conclusions about the differences between the parent metal, HAZ, and filler metal were made. It also allowed differentiating metallographic structures formed after welding and after the heat treatments. Figure 4, Figure 5, Figure 6 and Figure 7 demonstrate the SEM analysis results of the selected samples. In Figure 4, with 500× magnification, it is possible to verify that the parent metal is located at the lower right-hand of the Figure 4a,b, while the filler metal is in the upper left-hand side of the images Figure 4a,b. In the diagonal, the HAZ and weld interface can be verified. In Figure 4a,b we observed that large nodules of graphite split themselves due to the thermal cycle and diffused them into the filler metal. This effect was also promoted by the filler metal used since this electrode can capture some of the existing carbon in the base material and precipitates it in the form of graphite. This happened mainly when two passes were used, because when four passes were performed, the filler metal does not reveal small nodules of graphite. Moreover, in the case of four passes, the HAZ is narrower and fainter. Figure 5 shows the boundary zone between parent metal and HAZ, using a SEM magnification of 1000×. In Figure 5a, it is possible to observe large needle-shape structures and small graphite nodules in the HAZ area. This image verifies the present of a gradual transition of the filler metal indicated by a slender band combined with the weld interface. In Figure 5b, a sudden interface transition is noticeable, as well as the carbon diffusion process which is illustrated by the carbon impoverishment surrounding the larger graphite nodules (light dyed circles). Figure 6c also illustrates the carbon diffusion process in the HAZ which is characterized by the small black circles in the image and the large dispersion of small-sized graphite spherulites in the HAZ area. In Figure 6a and Figure 7a, the presence of ledeburite (needle-shaped structures) and small amounts of martensite, especially in the HAZ and in the PMZ area, are shown. 

At 2500× magnification, the needle-shaped structure seen on samples 1P0W2P400 and 2P300W2P300 is similar to the ledeburite structure. This structure is characterized as being of high wear resistance and hardness. It is also known to be harmful to welded joints due to its inability to absorb residual stresses. Ledeburite is formed by the fast cooling after the contact with the electric arc during the welding process. By viewing Figure 7a–c at 10,000× magnification, it is possible to observe the presence of martensite and cementite. Similar to ledeburite and cementite, martensite also has harmful characteristics for the welded joint such as hardness and brittleness, which affects the mechanical strength under tensile stress, therefore, limiting the plastic deformation and promoting the sudden fracture of the samples. Despite this, these samples revealed mechanical strength above the lowest acceptable limit, showing that the heat treatments imposed to the welds are options to be considered.

### 3.3. Hardness Test Results

**Figure 8:**
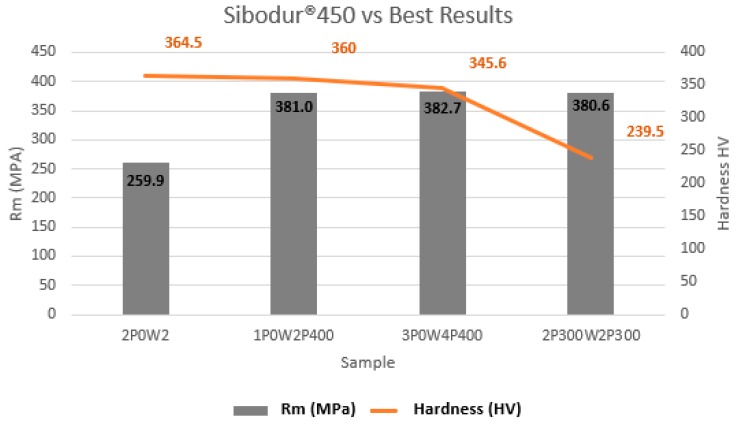
Comparison of hardness in the HAZ and UTS of the samples 2P0W2P0, 1P0W2P400, 1P0W4P400, and 2P300W2P300.

**Figure 9:**
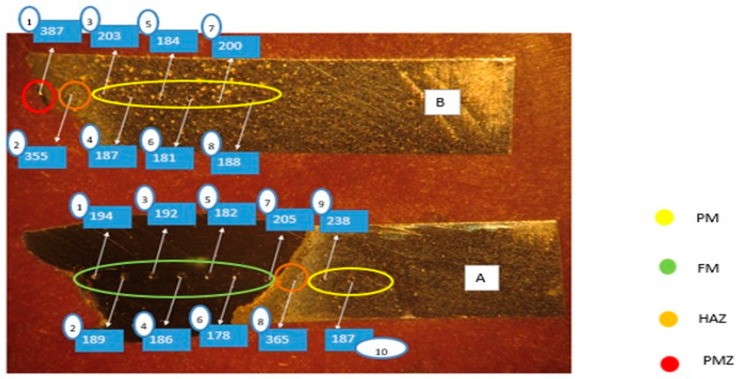
Indentations in sample 1P0W2P400 (PM–parent metal, FM–filler metal, HAZ–heat-affected zone, PMZ–partially melted zone) (values in HV).

According to [36] standards, a line of indentations was executed along the entire surface of the experimental samples. Figure 8 shows a comparison between the sample which was welded and did not suffer any heat treatment, and the best samples that occurred in this study. Considering the sample 1P0W2P400 as the best result, Figure 9 shows the demonstration of the indentations that were made, and how the hardness values were calculated for each area: PMZ, HAZ, FM, and MB.

**Figure 10:**
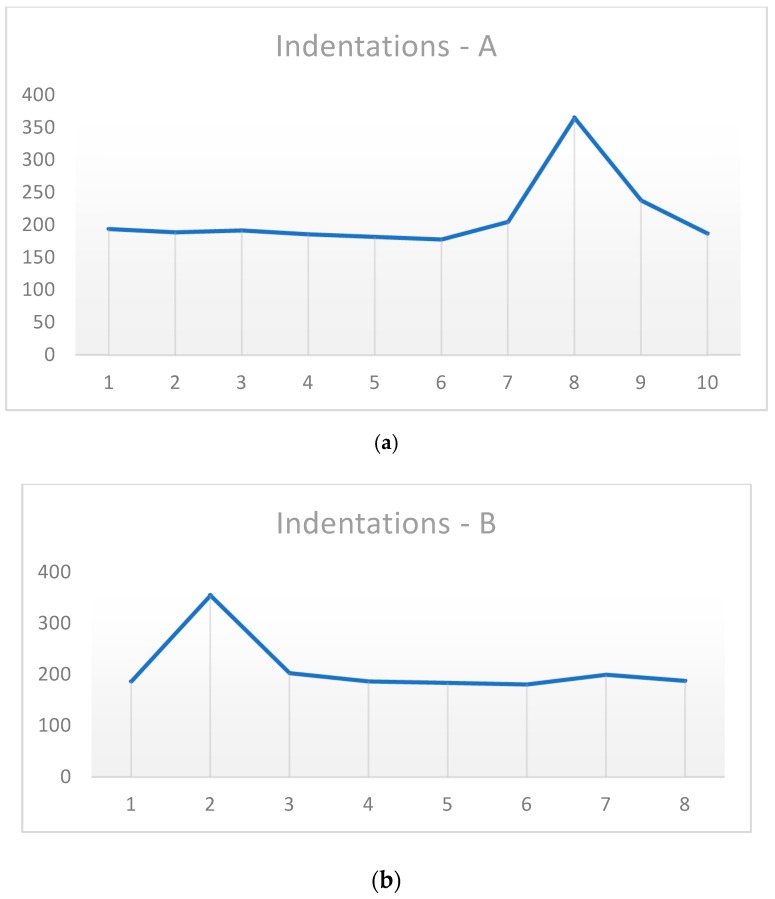
Indentations on the sample 1P0W2P400 after rupture: (**a**) side A and (**b**) side B.

It is possible to verify in Figure 8 that heat treatments are necessary to obtain satisfactory mechanical properties in the welded joint. By comparing sample 2P0W2P0 (no heat treated) with the other three samples, we observed that there is a decrease in hardness values at the HAZ area, mainly in the sample 2P300W2P300. On the basis of observations from Figure 10, we conclude that the hardest areas are PMZ (387 HV) and then HAZ (365–355 HV), due to the existence of the ledeburite and the martensite phases. In addition, the parent metal hardness (238–187) is superior to the filler metal hardness (194–178 HV).

## 4. Discussion

**Figure 11:**
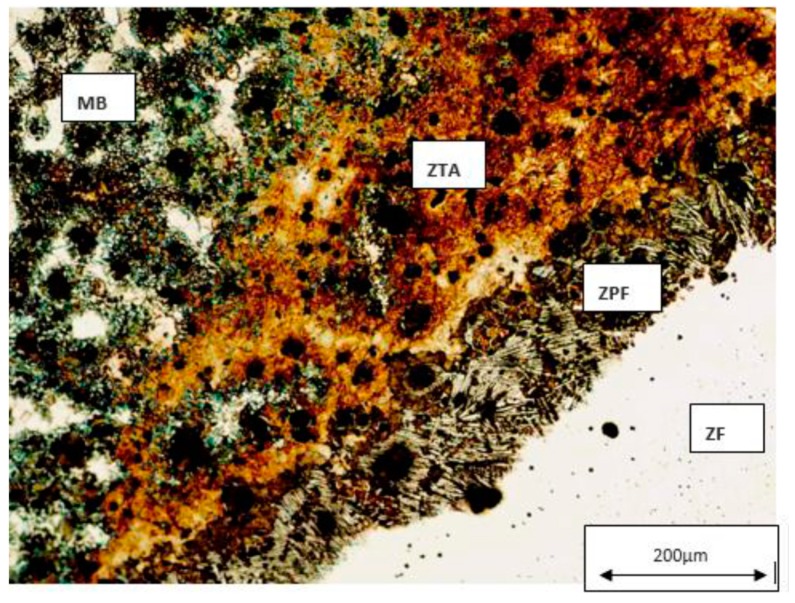
Metallographic analysis of the 2P0W2P0 sample, with 4% Nital and 200× magnification.

The sample 1P0W2P400 was considered the best sample in this study due to its UTS value, and less consumption of energy, time, and costs. This sample has a UTS value of 380.0 MPa, which correspond to 84% of the UTS value of the original parent metal (manufacturer’s specification). Figure 11 represents the metallographic analysis of the 2P0W2P0 sample which is in a raw state. This sample presents a UTS of 255.0 MPa, which corresponds to 56.7% of the parent material UTS. Thus, this type of cast iron really needs heat treatment to improve its mechanical properties on the welded joints.

The 1POW2P400 sample was submitted to a PWHT at 400 °C for 2 h with two passes which seemed to promote good mechanical properties, as well as the experimental sample 1P0W4P400 that was subjected to a PWHT at 400 °C for 2 h but it was welded with four passes. Both samples had approximately the same UTS results, and therefore it is possible to conclude that performing welding with two or four passes does not have a significant outcome when subjecting the samples to a PWHT at 400 °C for 2 h. Therefore, a PWHT cycle of 400 °C for 2 h with only two passes is more favorable, as the welding process is easier and less time-consuming. The same comparison can be done with the sample 2P300W2P300, which spends one more hour in energy when preheating, and since the material is very soft, the welding procedure to obtain a perfect bead is lagging and hard to achieve.

By comparing the samples 1P0W4P400 and 1P0W2P400 (Figure 6a,c), a noticeable difference in terms of quantity of martensite is seen, which is demonstrated by the smaller graphite nodules and lower formation of this structure in the welded zone. This evidence is the justification for the good results achieved in [33] with the experimental sample P300_N_P300_W4_P0. Table 3 shows the most significant results obtained in [33] which served as the basis for this study.

Furthermore, as previously cited, the martensite structure is prejudicial for welded joints, due to its hardness and brittleness nature, which contributes to a decrease in the material ductility, and consequently, a minor presence of this structure is advantageous. 

In the results obtained on [34], the parameters are different as compared with those used in this study. First, the filler metal is different, which probably is the main reason why sample P300_N_P300_W4_P0 had a similar UTS result as compared with the UTS value of the base material and much superior to 2P300W2P300. It was already tested that there is no significant change in welding with two or four passes, and also by comparing the sample 2P300W2P300 with P300_N_P300_W2_P0, which had the similar preheating temperatures and the same number of passes. Thus, Inconel really helped to enhance the better UTS results of the welded joint. 

On the other hand, these procedures and the filler metal, are much more expendable than those presented in this study. With a cheaper filler metal and less energy consumption, it is possible to obtain values close to those obtained in the sample P300_N_P300_W2_P0 in the previous study.

On the basis of the hardness test results it was possible to verify that higher hardness values were clustered in the PMZ and HAZ areas. When high hardness values are achieved it is a denotation that structures with high brittleness and lower ductility are present. This can lead to easier fracture formation and, consequently, part failure, which was present in the results of the tensile tests, since all experimental samples failed on the HAZ area, where larger concentrations of martensite and ledeburite were present.

## 5. Conclusions

On the basis of the results obtained, it is possible to claim that the main goal was achieved. An average UTS value of 380 MPa was reached when welding SiboDur^®^ 450 with E C Ni-Cl (EN ISO 1071:2015) as the filler metal. This corresponds to an average decrease of 15.5% in UTS as comparing with the UTS value of the parent metal.

In this study, three heat treatments, PWHT of 400 °C with two and four passes for two hours, and preheat at 300 °C for one hour combined with PHWT with the same temperature for two hours, with two passes, were carried out and all of them presented similar results. This study verifies that the heat treatment that better fits the industrial needs is the PWHT of 400 °C for 2 h using two passes, due to the energy, cost, and time savings, and consequently, its productivity. Thus, it is possible to use welding procedures with high strength DCIs for repair applications while still maintaining good mechanical properties.

From the results, we conclude that it is viable to obtain UTS values on the weld joint above 80% of the UTS value of the parent metal. Therefore, it is possible to verify that these heat treatments had similar results and in summary:

- It was achievable to generate better mechanical properties on the weld joint by applying heat treatments;
- The quantity of martensite and ledeburite decreased substantially as compared with the raw sample;
- Heat treatments such as PWHT and preheating combined with PWHT are beneficial to the welded joint;
- Increasing the number of passes increases the mechanical properties of the welded joint, however, it does not have a very significant impact.

Considering the previous work [34], this study has extended our knowledge about the weldability of these high strength cast irons, allowing us to state that:

- The mechanical strength of the welded joints produced with Ni filler metal is slightly lower (−15.5% related to the parent metal) than the results obtained using Inconel as filler metal (–4.4%). Moreover, the microstructure images show that the Ni filler metal allow greater carbon diffusion than the Inconel filler metal;
- Contrary to the previous study, PWHT showed better results than preheating treatment. This is mainly owing to the decrease of hard structures in the HAZ and PMZ, such as martensite and ledeburite, which contributes to the increase of hardness and brittleness, favoring the nucleation and propagation of cracks which lead to the failure of the welded samples;
- Using the preheating temperature used in the previous work, the only combination able to produce good results is to carry out a PWHT at 300 °C immediately after the welding, which resulted in an UTS slightly higher than 380 MPa. Higher PWHT temperatures lead to worse results;
- As in the previous work, an increase in the number of passes induces better results in terms of mechanical strength. However, this alternative implies the use of more energy, and the improvement in the mechanical strength is clearly residual, and it does not advise the use of more than two passes.

## Figures and Tables

**Table 1:** Chemical composition of Sibodur^®^ 450.

	C	Si	Mn	Cu
Chemical composition (%)	3.3–3.7	2.6–2.8	0.05–0.3	0.05–0.3

**Table 2:** Differences between the welding procedures and heat cycles parameters and tensile test obtained results.

Sample Code	PreHeating Treatment Cycle	E C Ni-Cl Welding	Post-Heat Treatment Cycle	UTS (MPa)	Average UTS (MPa)	Standard Deviation (MPa)	UTS Experiment/UTS Sibodur^®^ 450 (%)
1P0W2		80 A/2 passes		261.6	259.9	4.4	57.8
2P0W2				255.0			
3P0W2				263.2			
1P300W2	300 °C v/2 h	70 A/2 passes		356.8	354.3	4.0	78.7
2P300W2				349.7			
3P300W2				356.4			
1P0W2P400		80 A/2 passes	400 °C/2 h	390.0	381.0	10.8	84.7
2P0W2P400				369.0			
3P0W2P400				384.0			
1P0W2P500		80 A/2 passes	500 °C/2 h	375.1	370.0	8.3	82.2
2P0W2P500				360.6			
3P0W2P500				374.6			
1P0W2P700		80 A/2 passes	700 °C/2 h	335.1	346.2	10.6	76.9
2P0W2P700				356.4			
3P0W2P700				347.0			
1P0W4P400		60 A/4 passes	400 °C/2 h	394.0	382.7	25.0	85.0
2P0W4P400				354.0			
3P0W4P400				400.0			
1P0W4P500		60 A/4 passes	500 °C/2 h	337.5	360.2	22.9	80.1
2P0W4P500				359.9			
3P0W2P500				383.3			
1P0W2P300		80 A/2 passes	300 °C/2 h	357.0	375.4	16.6	83.4
2P0W2P300				380.0			
3P0W2P300				389.3			
1P300W2P300	300 °C/1 h	70 A/2 passes	300 °C/2 h	361.3	380.6	17.2	84.6
2P300W2P300				394.2			
3P300W2P300				386.2			
1P300W2P400	300 °C/1 h	70 A/2 passes	400 °C/2 h	334.9	319.5	22.7	71.0
2P300W2P400				330.1			
3P300W2P400				293.5			
1P300W2P500	300 °C/1 h	70 A/2 passes	500 °C/2 h	343.5	311.0	34.4	69.1
2P300W2P500				275.0			
3P300W2P500				314.6			

**Table 3:** Best mechanical results obtained in study [34].

P300_N_P33_W4_P0	P300_N_P300_W2_P0	P300_N_P300_W2_P0
448.0 MPa	389.8 MPa	390.7 MPa

## References

[1] Davis J.R. (1996). Cast Irons: ASM Specialty Handbook.

[2] Santos J., Gouveia R.M., Silva F.J.G. (2017). Designing a new sustainable approach to the change for lightweightmaterials in structural components used in truck industry. J. Clean. Prod..

[3] Konecná R., Nicoletto G., Bubenko L., Fintová S. (2013). A comparative study of the fatigue behavior of two heat-treated nodular cast irons. Eng. Fract. Mech..

[4] Fragassa C. (2017). Material selection in machine design: The change of cast iron for improving the high-quality in woodworking. J. Mech. Eng. Sci..

[5] Mullins D.J. (1993). Engineering Properties, Specifications and Physical Constants of Specific Ductile Irons. Ductile Iron Handbook.

[6] De Santis A., Di Bartolomeo O., Iacoviello D., Iacoviello F. (2004). Quantitative shape evaluation of graphite particles in ductile iron. J. Mater. Process. Technol..

[7] Panneerselvam S., Putatunda S.K., Gundlach R., Boileau J. (2017). Influence of intercritical austempering on the microstructure and mechanical properties of austempered ductile cast iron (ADI). Mater. Sci. Eng. A.

[8] Fragassa C., Minak G., Pavlovic A. (2016). Tribological aspects of cast iron investigated via fracture toughness. Tribol. Ind..

[9] Vilela F. (2009). Uma Visão Sobre A Produção de Ferro Nodular. Fundição Rev. Assoc. Fundição Port..

[10] Dorazil E. (1991). High Strength Austempered Ductile Iron.

[11] Trudel A., Gagne M. (1997). Effect of Heat Treatment Parameters on the Characteristics of Austempered Ductile Irons.

[12] Sohi M.H., Ahmadabadi M.N., Vahdat A.B. (2004). The role of austempering parameters on the structure and mechanical properties of heavy section ADI. J. Mater. Process. Technol..

[13] Harding R.A. (2007). The production, properties and automotive applications of austempered ductile iron. Kov. Mater..

[14] Amaral H.M.F.M. (1989). Soldadura dos ferros fundidos cinzentos, nodulares e bainíticos. MSc. Thesis.

[15] Modenesi P.J., Marques P.V., Bracarense A.Q. (2005). Soldagem-Fundamentos e Tecnologia.

[16] Pascual M., Cembrero J., Salas F., Pascual-Martínez M. (2008). Analysis of the weldability of ductile iron. Mater. Lett..

[17] El-Banna E.M. (1999). Effect of preheat on welding of ductile cast iron. Mater. Lett..

[18] Mirhedayatian S.M., Vahdat S.E., Jelodar M.J., Saen R.F. (2013). Welding process selection for repairing nodular cast iron engine block by integrated fuzzy data envelopment analysis and TOPSIS approaches. Mater. Des..

[19] Ebrahimnia M., Ghaini F., Gholizade S., Salari M. (2012). Effect of cooling rate and powder characteristic on the soundness of heat affected zone in powder welding of ductile cast iron. Mater. Des..

[20] Bęczkowski R. (2017). Repair welding of the massive cast. Arch. Foundry Eng..

[21] Pouranvari M. (2010). On the weldability of grey cast iron using Nickel based filler metal. Mater. Des..

[22] Sun D.Q., Gu X.Y., Liu W.H., Xuan Z.Z. (2005). Welding consumable research for austempered ductile iron (ADI). Mater. Sci. Eng. A.

[23] Ghaini F.M., Ebrahimnia M., Gholizade S. (2011). Characteristics of cracks in heat affected zone of ductile cast iron in powder welding process. Eng. Fail. Anal..

[24] Gouveia R.M., Silva F.J.G., Paiva O.C., Andrade M.F., Pereira L.A., Moselli P.C., Papis K.J.M. (2018). Comparing the Structure and Mechanical Properties of Welds on Ductile Cast Iron (700 MPa) under Different Heat Treatment Conditions. Metals.

[25] Askary-Paykani M., Shayan M., Shamanian M. (2014). Weldability of Ferritic Ductile Cast Iron Using Full Factorial Design of Experiment. J. Iron Steel Res. Int..

[26] Mandal N.R. (2017). Ship Construction and Welding.

[27] El-Banna E., Nageda M., El-Saadat M. (2000). Study of restoration by welding of pearlitic ductile cast iron. Mater. Lett..

[28] Arabi-Jeshvaghani R., Shamanian M., Jaberzadeh M. (2011). Enhancement of wear resistance of ductile iron surface alloyed by stellite 6. Mater. Des..

[29] Rodriguez P. (2013). Manual de Soldadura.

[30] Yamamoto S. (2008). Arc Welding of Specific Steels and Cast Irons.

[31] Kumar R., Kumar M., Trivedi V., Bhatnagar R. (2017). Evaluation of Mechanical and Microstructural Properties of Cast Iron with Effect of Pre Heat and Post Weld Heat Treatment. SSRG Int. J. Mech. Eng..

[32] Connor L.P. (1987). Welding technology. Welding HandBook.

[33] Gouveia R., Silva F., Paiva O., Andrade M., Silva L., Moselli P., Papis K. (2017). Study of the Heat-Treatments Effect on High Strength Ductile Cast Iron Welded Joints. Metals.

[34] (2012). ISO 4136:2012—Destructive Tests on Welds in Metallic Materials—Transverse Tensile Test.

[35] (2003). ISO 17639:2003—Destructive Tests on Welds in Metallic Materials—Macroscopic and Microscopic Examination of Welds.

[36] (2011). ISO 9015-1:2011—Destructive Tests on Welds in Mmetallic Materials. Hardness Testing. Hardness Test on Arc Welded Joints.

[37] (2019). DIN EN 1563:2019—Founding—Spheroidal Graphite Cast Irons; English translation of DIN EN 1563:2012-03.

[38] Automotive G.F. (2015). Ductile Iron: SiboDur^®^ and GJS family. Material DataSheets, Research and Development.

[39] (2015). EN ISO 1071: 2015—Welding Consumables -- Covered Electrodes, Wires, Rods and Tubular Cored Electrodes for Fusion Welding of Cast Iron – Classification.

